# Perceiving actions before they happen: psychological dimensions scaffold neural action prediction

**DOI:** 10.1093/scan/nsaa126

**Published:** 2020-09-28

**Authors:** Mark A Thornton, Diana I Tamir

**Affiliations:** Department of Psychological and Brain Sciences, Dartmouth College, Hanover, NH 03766, USA; Department of Psychology and Princeton Neuroscience Institute, Princeton University, Princeton, NJ 08540, USA

**Keywords:** action, predictive coding, social cognition, naturalistic fMRI, decoding

## Abstract

The social world buzzes with action. People constantly walk, talk, eat, work, play, snooze and so on. To interact with others successfully, we need to both understand their current actions and predict their future actions. Here we used functional neuroimaging to test the hypothesis that people do both at the same time: when the brain perceives an action, it simultaneously encodes likely future actions. Specifically, we hypothesized that the brain represents perceived actions using a map that encodes which actions will occur next: the six-dimensional Abstraction, Creation, Tradition, Food(-relevance), Animacy and Spiritualism Taxonomy (ACT-FAST) action space. Within this space, the closer two actions are, the more likely they are to precede or follow each other. To test this hypothesis, participants watched a video featuring naturalistic sequences of actions while undergoing functional magnetic resonance imaging (fMRI) scanning. We first use a decoding model to demonstrate that the brain uses ACT-FAST to represent current actions. We then successfully predicted as-yet unseen actions, up to three actions into the future, based on their proximity to the current action’s coordinates in ACT-FAST space. This finding suggests that the brain represents actions using a six-dimensional action space that gives people an automatic glimpse of future actions.

The social world is abuzz with people doing things. People are constantly talking, eating, working, playing, walking, jumping, snoozing and so on. In order to navigate the social world, one needs to be able to accurately recognize and understand others’ current actions (Thornton and Tamir, 2019b). However, to truly succeed in the social world, one also needs to predict others’ future actions. To help a friend achieve their goals, one must understand the sequence of actions they will take to reach those goals; to hinder an enemy, one must likewise predict their upcoming moves.

People can draw upon many types of knowledge to inform such action predictions. Knowing what personality traits a person possesses, what mental state they currently occupy, or what situation they find themselves in could all inform inferences about their likely future actions (Abbott *et al.*, 1985; Gilbert and Malone, 1995; Frijda, 2004; Gibson, 2014). While these sources of information are well-studied predictors of actions, here we examine another source of information about people’s future actions: their current actions.

We propose that the brain automatically predicts others’ future actions while perceiving their current actions. The human perceptual system constantly engages in this kind of reflexive prediction. While watching a ball fly through the air, people can simultaneously recognize its current location and predict its future trajectory, thereby allowing them to turn their back on it momentarily and still run to the right spot to catch it. The theory of predictive coding suggests that when people perceive the world, their brains do not merely process and encode incoming information (Rao and Ballard, 1999). Instead, they make predictions about the likely future, and continually compare these predictions against new sensory data. These continual predictions help to fill in gaps in one’s perception and allow us to plan our actions by anticipating future states of the world (Knill and Pouget, 2004; Friston and Kiebel, 2009; Friston, 2010, 2012).

Researchers originally tested the predictive coding hypothesis in the domains of basic sensation, such as vision and audition (Hohwy *et al.*, 2008; Vuust *et al.*, 2009). More recently, researchers have found that the same principle applies to social cognition as well (Barrett and Bar, 2009; Koster-Hale and Saxe, 2013; Barrett and Simmons, 2015; Theriault and Young, 2017; Tamir and Thornton, 2018; Thornton *et al.*, 2019). This research suggests that social cognition is fundamentally organized around the goal of predicting other people’s mental states and behaviors. Mirror neurons offer an example of this principle. These neurons were originally identified based on their responsiveness to both perceived and performed actions (Rizzolatti and Craighero, 2004; Keysers and Gazzola, 2010; Oosterhof *et al.*, 2013). That is, the same neuron might spike when picking up a glass oneself or watching someone else pick up a glass. However, recent studies suggest that mirror neurons do more than represent current action: they also activate predictively, right before an action occurs (Kilner *et al.*, 2007; Saygin *et al.*, 2011; Maranesi *et al.*, 2014).

By linking perceptual and motor systems, mirror neurons offer a compelling mechanism to explain how we might understand the actions others are currently performing as well as the actions they are soon to perform. However, mirror neurons are not sufficient to grant perceivers predictive insight deep into the social future. To achieve deep foresight, perceptions of a person’s current actions must be combined with broader conceptual knowledge of actions. For example, one’s perceptual system might determine that another person is currently running; mirror neurons might help one predict the sequence of motor actions that will allow them to take their next steps. However, these systems cannot predict what actions the runner will take after they stop running. To make that prediction, one must draw upon their knowledge about running. For instance, one might know that running makes people sweaty, and sweaty people like to shower, and therefore, one can predict that the runner will shower in the near future. Thus, conceptual knowledge must complement perceptual information for perceivers to make deeper predictions about the actions of other agents.

If deep action prediction relies on conceptual knowledge, then what do we know about people’s conceptual knowledge of actions? Recent work suggests that people organize their conceptual knowledge of using a low-dimensional representational space (Tamir *et al.*, 2016; Tamir and Thornton, 2018; Thornton *et al.*, 2019). That is, people do not need to independently represent all of the nuances of each and every action. Instead, the brain can distill much of its representations of actions to coordinates on just a few psychologically meaningful dimensions. Specifically, when people think about actions, they extract information about that action on six dimensions: the Abstraction, Creation, Tradition, Food(-relevance), Animacy and Spiritualism Taxonomy (ACT-FAST; Table 1; Thornton and Tamir, 2019b). Each action occupies a single coordinate in this six-dimensional action space. By knowing where an action falls on each of these dimensions, a person can efficiently represent another person’s current action. For example, consider the action of ‘designing a wedding cake’. This action is high (i.e. near ‘Pole 1’ in Table 1) on all six dimensions: designing something is an abstract activity, and weddings are social; it involves creating something; it is a highly traditional type of activity, associated with a long historical tradition; it obviously involves food; it is an action performed by an animate agent (a baker) rather than a natural force or machine and it is relevant to people’s spiritual lives, as weddings are often religious or take on transcendent meaning.

**Table 1.** Dimensions of ACT-FAST

Dimension	Pole 1	Pole 2	Examples
Abstraction	Abstract/social	Concrete/physical	Govern, refute *vs* drip, peel
Creation	Creation	Crime	Film, sing *vs* prosecute, testify
Tradition	Tradition	Innovation	Cook, decorate *vs* emit, encrypt
Food	Food	Non-food	Bake, fry *vs* detain, testify
Animacy	Animate	Mechanical	Meow, floss *vs* contain, extract
Spiritualism	Work	Worship	Fax, haggle *vs* foretell, ascend

We propose that this particular organization of action knowledge is attuned, specifically, to action prediction. That is, actions are located in this space close to other actions that they are likely to predict or follow; conversely, actions that are far away from each other are unlikely to precede or follow each other. Proximity on the ACT-FAST dimensions can thus be used to guide action prediction. For example, the actions ‘eating’ and ‘cooking’ are both Food-relevant words, and thus located close to each other on this dimension of ACT-FAST. This proximity reflects the semantic association between these actions, but it does so as a byproduct of their transition likelihood: if someone is currently cooking, they are likely to soon starting eating. This principle of proximity predicting transitions already been demonstrated in the domain of mental states (Tamir *et al.*, 2016; Thornton *et al.*, 2019), and we suggest that it can help explain action prediction as well.

This model is useful as a predictive tool only to the extent that it accurately captures the statistical regularities of action sequences that occur in the natural world. Such statistical regularities underlie many forms of learning. For example, language learning depends on the mind’s ability to track the transitional probabilities between units of speech (Saffran *et al.*, 1996). This allows us to predict the next phoneme in a word or the next word in a sandwich. However, many statistical regularities are irrelevant to prediction. All gerunds may end in ‘ing’, but this does not mean that one gerund predicts the next. In the domain of actions, a successful model of action representations should capture specifically those properties that allow for prediction. Whereas the motor act of putting a dish in the dishwasher superficially resembles putting food in the oven, our representation of actions would be better served by representing baking more similarly to frying than to dishwashing. If we find that the main dimensions that people use to make sense of actions do have this predictive property, it would suggest that action knowledge is indeed organized around the goal of prediction. Encoding actions in such a way—in a space where proximity reflects prediction—offers a highly efficient way to represent these regularities. A brain tuned to prediction should likewise take advantage of these predictive features when encoding actions.

In the present investigation, we use neuroimaging to test whether the brain automatically predicts others’ actions by encoding them on the ACT-FAST dimensions. Two empirical planks are necessary to support this hypothesis. First, when people perceive actions, their brain activity must spontaneously reflect the ACT-FAST dimensions. If so, we should be able to decode the location of actions on ACT-FAST dimensions based on patterns of brain activity. Second, the observed sequence of actions must move smoothly through ACT-FAST space. If so, we should be able to predict the likelihood of future actions based on how close they are to the current actions on the ACT-FAST dimensions.

To test these hypotheses, we used open functional magnetic resonance imaging (fMRI) data to examine participants’ brain activity as they watched a naturalistic video stimulus in the scanner. In order to know what actions participants were seeing, we annotated the actions occurring in this video using a deep learning algorithm. We then constructed a multivoxel predictive model of neural (i.e. blood-oxygen-level-dependent [BOLD] response) activity, which could decode participants’ current location in the six-dimensional action space. Using the principle of proximity, we tested whether a participant’s current location in this multidimensional action space predicted which actions actually occurred later in the video.

## Methods

The data and code from this study are freely available online at the Open Science Framework (https://osf.io/5xykq/).

### fMRI data

To examine the hypothesis that neural representations of current actions predict actual future actions, we drew upon open data from a previous investigation. This study collected functional neuroimaging data from participants’ brains as they watched a naturalistic video stimulus (Chen *et al.*, 2017). The data are publicly available on the Princeton DataSpace (http://arks.princeton.edu/ark:/88435/dsp01nz8062179). In the original study, a sample of 22 participants (12 male, 10 female, aged 18–26 years, mean age = 20.8 years) who had previously not viewed the television show Sherlock were recruited to watch half of the first episode of the series in the fMRI scanner. Five participants were excluded—two for head motion, two for poor recall of the movie and one for falling asleep—leaving a final sample of 17. Participants viewed the video in two segments of 23 and 25 minutes. Each segment was preceded by a cartoon of 30 s unrelated to the rest of the video (‘Let’s All Go to the Lobby’). During each viewing period, participants were asked to attend to the video, with no behavioral responses required. After the video, participants engaged in a verbal recall procedure. We did not use the recall data in the present investigation, so we will not discuss it further.

Imaging data were acquired using a 3T Siemens Skyra scanner with 20 channels head coil. Functional images were acquired from the whole brain (repetition time (TR) = 1.5 s, echo time (TE) = 28 ms, flip angle = 64°, 27 ascending interleaved slices of 4 mm thickness, 3 mm^2^ in-plane resolution, 192 × 192 mm field of view (FOV)). Anatomical images were obtained using a T1 magnetization prepared - RApid gradient echo (MPRAGE) protocol with 0.89 mm^3^ voxels. In the present investigation, we used preprocessed data from the original study. This preprocessing was carried out using FSL (http://fsl.fmrib.ox.ac.uk/fsl) and included slice-time and motion corrections, linear detrending, high-pass (140 s cutoff) filtering, linear spatial normalization to a common template brain (MNI152), spatial resampling to 3 mm isometric voxels, z-scoring over time per voxel, and 6 mm full width at half maximum Gaussian smoothing. The fMRI time-course was also shifted by 3 TRs (4.5 s) to align functional activity with the movie time stamps, accounting for hemodynamic lag.

### Automated action annotation

**Fig. 1.**
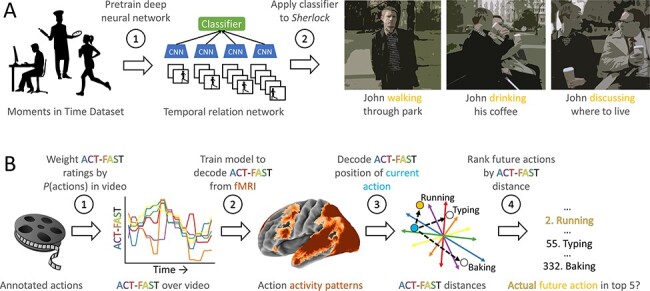
Analysis schematic. (A) Actions in Sherlock were automatically annotated using a temporal relation network pre-trained on the Moments in Time dataset. (B) These annotations were combined with ratings of where the actions fell on the ACT-FAST dimensions to produce an ACT-FAST time-series over the course of the video. These time-series were used to train a model to decode ACT-FAST coordinates from patterns of brain activity. We then created a rank order list of future actions, from most likely to least likely, ordered based on the proximity between each action in ACT-FAST space and the decoded coordinates. Accuracy was assessed by examining whether actual future actions appeared among the five ranked most likely.

The first step in the analysis process (Figure 1A) was to determine which actions participants were observing at each time point in the video. We identified the actions present in Sherlock at each moment using an automatic annotation tool. Specifically, we used a temporal relation network—a type of deep neural network classifier (Zhou *et al.*, 2017)—that was trained to identify actions in video. It was pre-trained on the Moments in Time Dataset (Monfort *et al.*, 2018), which consists of 1 million videos, each 3 s in length, representing a total of 339 different classes of actions. We split the video into 3 s segments to match the length of actions in the training set. We used non-overlapping segments to ensure that each action classification was performed on separate video data. The algorithm estimated the probability that each of the 339 actions occurred in each 3 s segment. We subsequently averaged together several actions that differed only in the agent performing them (e.g. male singing and female singing) ultimately producing estimates for the likelihood of 332 distinct actions across the entire Sherlock video.

### Action ratings

We hypothesized that the brain represents actions using a set of six psychologically relevant dimensions (‘ACT-FAST’, Table 1). To test whether participants in this study used these dimensions to represent the actions in the Sherlock video, it was necessary to first locate each of the 332 actions on each ACT-FAST dimension. To do so, we asked participants to rate each action on each dimensions. A subset of 46 of these actions had already been rated in prior work (Thornton and Tamir, 2019b), so we collected new data for the remaining 280 actions. We recruited participants (*N* = 662) using Amazon Mechanical Turk and TurkPrime (Litman *et al.*, 2017) to rate the actions. We excluded 16 participants for indicating non-native and less than excellent English proficiency, and an additional 46 participants providing 10 or fewer unique responses, leaving a final sample of 600 (294 female, 301 male, 2 other, 3 preferred not to indicate gender; mean age = 36.2, range = 18–69). All participants provided informed consent in a manner approved by the Princeton University Institutional Review Board.

Each participant was randomly assigned to rate actions on one of the six ACT-FAST dimensions. They were then randomly assigned to rate 70 actions on that dimension. The six dimensions and their poles were described to participants using definitions validated in an earlier study (Thornton and Tamir, 2019b). At the end of the survey, participants provided their demographic information. Following data collection, we averaged ratings across participants to provide a single set of ratings for each of the 280 actions on each of the ACT-FAST dimensions. We then combined these ratings and the previously existing ratings to locate each of 332 possible actions on each of the ACT-FAST dimensions. Average ratings were z-scored across actions on each dimension.

### Training a neural model of action representation

With all actions located in action space, we next tested whether the brain automatically uses this action space to represent and predict actions. To do so, we developed a statistical model to decode the coordinates of each action from neural data (Figure 1B). All steps of the analysis were conducted using bi-cross-validation scheme. That is, we divided the Sherlock video into five continuous sections of approximately equal length, based on DVD scene boundaries. We then used four-fifth of the movie from 16 of 17 participants as training data. The remaining one-fifth of the movie in the remaining participant was held out for testing. This procedure ensured that results generalize to both unseen video and new participants.

**Fig. 2.**
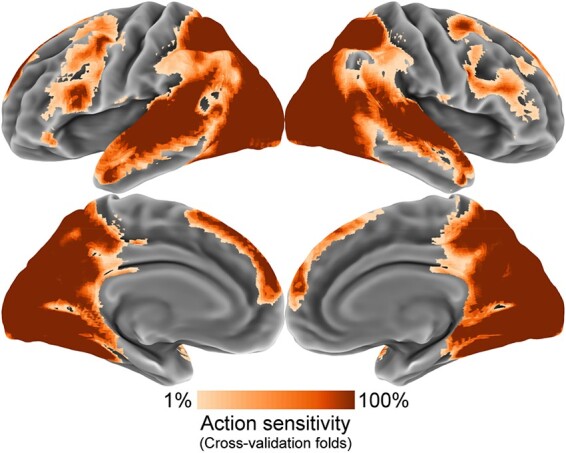
Feature selection results. To train and test the neural action prediction model, we selected voxels sensitive to action. We computed the reliability of each voxel’s action-specific activity across cross-validation folds. We then clustered voxels into action sensitive and non-sensitive classes based on these reliability values. The displayed results indicate the overlap of the selected voxels across cross-validation folds, with more consistently selected voxels shown in darker orange.

Feature selection was used to confine all neural decoding analyses to a set of voxels selected for action-sensitivity. Average patterns of brain activity were computed for each of the 332 actions. We then computed the reliability (Cronbach’s α) across participants of each voxel’s activation to these actions. These voxel-wise reliabilities were then entered into a Gaussian mixture model to cluster high- and low-reliability voxels. Figure 2 indicates the voxels which were consistently selected by this procedure across folds of the cross-validation.

We next trained a decoding model to ‘read out’ patterns of brain activity within the feature-selected regions as coordinates on the ACT-FAST dimensions. This decoding model consisted of a set of six independent partial least-squares (PLS) regressions, one for each action dimension (McIntosh *et al.*, 1996). PLS regressions are an integrated factor analysis and regression technique. They take high-dimensional data, simplify it to a smaller number of components, and then use those components to predict a dependent variable. In this case, the high-dimensional data consisted of patterns brain activity within the action sensitive regions identified in the feature selection and the dependent variable was one of the six ACT-FAST dimensions.

We generated this dependent variable as follows: At each time point, we knew the likelihood of all 332 actions, based on the output of the automated annotation process. In addition, we knew the location of each action on each action dimension based on human ratings. We could thus determine the location of each time point in action space by averaging across the action dimension ratings, with each action’s ratings weighted by the likelihood that that action was on screen during that time point. For example, if several food-related actions were judged to be very likely at a given time point, and non-food related actions were unlikely, then the ultimate coordinate of that time point would be high on the Food dimension. For each of the six action time series generated in this way, we then trained a PLS decoding model. The only hyper-parameter that must be assigned for PLS regression is the number of components. We used nested split-half cross-validation to learn this parameter within each fold of the primary cross-validation. We visualize the PLS regression weights in Supplementary Figure S1.

### Computing reference coordinates

The decoding models fit in the previous section allow us to infer ACT-FAST coordinates from patterns of brain activity. Each of the 332 possible actions already had coordinates on the ACT-FAST provided by human rater. However, insofar as the decoding models were not perfectly accurate, the human-rated coordinates might differ from the model-fitted coordinates. Since subsequent analyses rely on the output of the decoding model, rather than human judgments, we therefore we extracted the fitted ACT-FAST values for each of the 332 possible actions from the neural patterns. To do so, we applied the trained PLS models to decode each TR of the training data. We then averaged these coordinates across the training portion of the video, weighting them by the probability of each action over time. This procedure yielded a set of neural ACT-FAST coordinates for use as a reference in the testing phase. They reflect where, in the action space, the brain places each one of the possible actions. Procrustes rotation was used to align these reference coordinates with each participants’ unique neural representational space, still using only the training portion of their data.

### Testing a neural model of action prediction

We next turned from our training data to our test data. Within the test portion of the video, we computed ‘discrete actions’ based on the action annotations. We defined a discrete action as a continuous run of TRs in which the same action class was judged to be most likely by the automated annotation. For example, if ‘eating’ had the highest probability for 10 TRs, then that 15 s section of the video would be considered a single discrete action. Discrete actions ranged in length from 3 to 39 s, with an average of 4.43 s. Our goal was to test whether the decoding model we had trained in separate data could successfully predict which discrete actions were on screen, or would soon be on screen, in the test data.

We then applied our trained decoding model to the held-out test data. This produced decoded ACT-FAST values for each TR in the test set. We averaged the decoded ACT-FAST values across TRs within each discrete action to produce a single set of coordinates for each discrete action. Next, we computed the distance from the decoded coordinates across that discrete action to the reference coordinates of each of the 332 possible actions. For example, one discrete action might have decoded coordinates very close to the reference position of ‘walking’ and very far from the reference position of another action like ‘eating’. We used these distances to rank the likelihood of each possible action, with closer actions (e.g. walking) regarded as more likely, and more distant actions (e.g. eating) regarded as less likely. Thus, the result was a rank-ordered list of all the actions, from most to least likely. We created a rank-ordered list of actions for the combined ACT-FAST model, using Euclidean distances between coordinates on all six dimensions at once, as well as the separately for each ACT-FAST dimension (e.g. just ‘Food’).

To measure the accuracy of the model’s predictions, we compared the rank-ordered list to the actions which actually occurred in the video. To do so, we use used a ‘top 5’ accuracy metric. If the actual action in the video was among the first five in the rank-ordered list, then the model was regarded as accurate in that particular instance. We assessed top 5 accuracy at six time points (Lags 0–5) relative to each discrete action. Lag 0 represented the current action itself. If the current action was among the first five in the rank-ordered list, then this was taken as affirmative evidence that the model accurately decoded the identity of action at Lag 0. We averaged across all instances of Lag 0 actions to get a measure of how well participants’ brains encode perceived actions on the ACT-FAST dimensions.

We conducted the same analysis to test how well participants’ brains predict actions using the ACT-FAST dimensions. Lag 1 represented the next discrete action after the current action, and Lag 2 represented the discrete action after that and so forth. If the next action was among the first five in the rank-ordered list, then this is taken as affirmative evidence that the model accurately predicted the identity of actions at these subsequent lags. We averaged across all instances of Lag 1 actions to get a measure of Lag 1 accuracy; we averaged across all instances of Lag 2 actions to get a measure of Lag 2 accuracy and so on. Accuracy at each subsequent lag indicate that the ACT-FAST representation of the current action also automatically encodes which other actions are likely to occur in the near future.

Importantly, these analyses never compare the neural patterns for one action with the neural patterns of any subsequent action. Nor do we compare the ACT-FAST coordinates decoded from the neural patterns for one action with the ACT-FAST coordinates decoded from the neural patterns for any subsequent actions. Rather, the coordinates of the decoded Lag 0 action are compared with the reference coordinates for all 332 possible actions (computed on data from a different portion of Sherlock). This comparison is used to make predictions for all subsequent lags, without ever decoding the ACT-FAST coordinates of those later actions. These predictions are then compared to the actual labels for the actions at each time point. As a result, these analyses cannot capitalize on autocorrelation in the BOLD signal itself, which might occur for a variety of reasons that have nothing to do with predictive coding. Thus, the model will only predict future actions better than chance if actions that are temporally proximal in Sherlock are also conceptually proximal in the ACT-FAST space, indicative of predictive coding.

To create a baseline against which to compare observed accuracy, we used permutation testing. Specifically, we permuted the 332 actions with one another, so that—for instance—‘running’ might take on the ACT-FAST coordinates of ‘singing’ in one permutation. We used these permuted coordinates to compute an empirical null distribution of the top 5 accuracy metric. The observed accuracies could then be compared against this null distribution to determine the statistical significance of model performance within individual participants, or the sample as a whole.

## Results

The ACT-FAST decoding model identified the actions that participants were currently viewing with above-chance accuracy. Across participants, the average top 5 accuracy at Lag 0 was 3.43%. This performance more than doubled the chance level performance (mean permutation accuracy) of 1.51%. Moreover, this performance was statistically significant not just at the group level (*P* = 0.0003), but also in 94% of the individual participants (16 of 17), indicating widespread accuracy in the sample. These results indicate that the brain spontaneously represents actions on the ACT-FAST dimensions during naturalistic viewing.

**Fig. 3.**
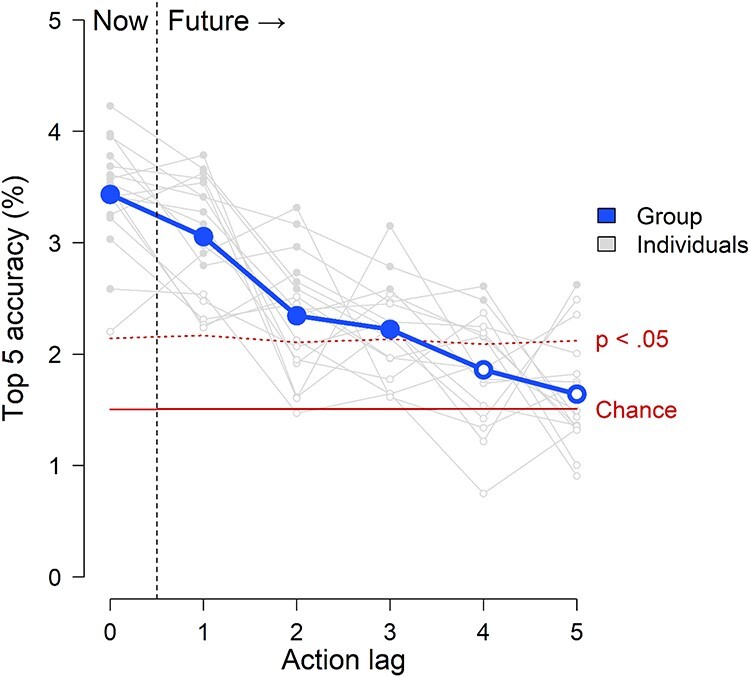
Combined ACT-FAST action prediction results. Coordinates in ACT-FAST space were decoded from brain activity. These decoded coordinates supported accurate classification of the actions which participants were currently viewing (Lag 0). Moreover, the decoded coordinates of the present action also predicted as-yet unseen actions later in the video (Lags 1–3).

The ACT-FAST decoding model also predicted future actions in the video, as-yet unseen by the participant with above-chance accuracy. The decoded ACT-FAST coordinates of each current discrete action predicted not only the identity of that action, but also up to three actions into the future (Figure 3). At Lag 1—the discrete action immediately following the one currently viewed—model accuracy was 3.06% (*P* = 0.0059). At Lag 2, accuracy was 2.35% (*P *= 0.022), and at Lag 3, accuracy was 2.22% (*P* = 0.036). However, by Lag 4, accuracy fell below the threshold of statistical significance at 1.86% (*P* = 0.13) and remained there at Lag 5, with 1.64% accuracy (*P* = 0.30). The average length of each action was 4.43 s, so the ability of the model to predict accurately up to Lag 3 suggests that the information embodied in the ACT-FAST representations of perceived actions could allow people to predict others actions up to about 13 s into the future, on average.

**Fig. 4.**
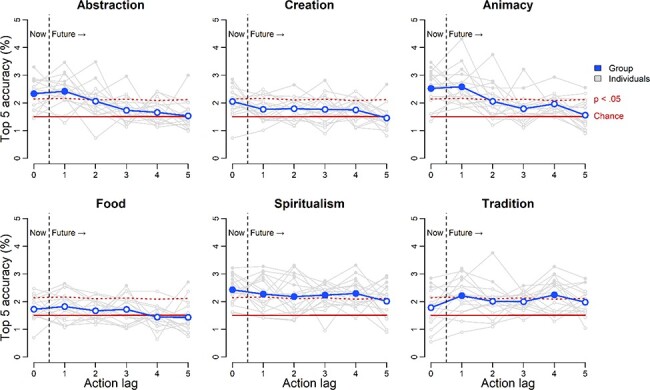
Action predictions from individual ACT-FAST dimensions. Distinct dimensions of the ACT-FAST support action representation and prediction. Abstraction, Animacy and Spiritualism all support accurate decoding of both current and future actions.

Results from the analysis of individual dimensions offer mixed evidence of predictive coding (Figure 4). Three dimensions—Abstraction, Animacy, and Spiritualism—were accurately decoded at Lag 0. Abstraction and Animacy were also accurate at Lag 1, and Spiritualism remained significantly accurate at the group level out to Lag 4. Tradition was intermittently accurate at Lags 1 and 4, but not others. Creation and Food did not achieve statistically significant performance at any time point. These results suggest that certain dimensions of the ACT-FAST may contribute more than others to representing likely future actions. On average, 6.67 neural components were necessary to achieve optimal decoding of the ACT-FAST dimensions in the PLS regressions. The dimensionality of the neural code varied across dimensions from 7.77 for Animacy to 5.54 for Food. Consistent spatial patterns encoded each dimension across the brains of different participants (Supplementary Figure S1).

## Discussion

In this study, people watched a naturalistic video rife with rich action sequences. We tested how people represented the current action they perceived on screen, and whether those representations could foretell future actions that people had not yet seen. Our findings suggest that people accomplish both of these by representing actions using a low-dimensional action taxonomy tuned for prediction. That is, the action space described by ACT-FAST provides a window into both perceived and future actions. We were able to decode positions in ACT-FAST space from patterns of brain activity as participants viewed actions in an episode of Sherlock. Moreover, by decoding ACT-FAST coordinates from current brain activity, we could predict actions as-yet unseen by participants. The closer a possible future action was to the present coordinates in ACT-FAST space, the more likely that action was to occur as one of the next three actions in the video. Three of the six ACT-FAST dimensions also significantly predicted future actions on their own. Together, these current findings provide evidence for the central role of prediction in action perception.

The ACT-FAST dimensions were developed to provide a comprehensive description of how people think about actions (Thornton and Tamir, 2019b). They reflect the conceptual structure of action knowledge. The present results suggest that people’s conceptual knowledge of actions may be functionally tuned for prediction. One can easily imagine dimensions of action representation which capture the useful features of action space but fail to capture the temporal dynamics of action sequences. For example, knowing which letter of the alphabet that an action word starts with is very useful. However, this information, like much of what we know about actions may help people to represent or perceive a current action, cannot predict which action will come next. Thus, not all relevant dimensions of action representation yield useful predictions about future actions. Indeed, here we do not observe that all six ACT-FAST dimensions predict future actions. However, this may stem from limitations in the stimulus, in which we examine actions over a short, fine-grained timescale. In other research, we have observed that different dimensions predicting action transitions at longer timescales (Thornton and Tamir, 2019a). However, the ACT-FAST as a whole appears uniquely suited to the goal of action prediction, outperforming more than 95% of statistically comparable sets of dimensions (see Supplementary data). Indeed, separate behavioral research suggests that people use ACT-FAST when making explicit predictions about how one action follows from another (Thornton and Tamir, 2019a). These findings raise the question of whether people’s conceptual understanding of actions may in fact be shaped by the goal of predicting them.

Actions are only one of multiple domains of social knowledge. Our theoretical framework for predictive social cognition suggests that multiple types of social knowledge—including mental states and traits—are all organized into low-dimensional representational spaces (Tamir and Thornton, 2018). Neural representations of mental states are organized by three dimensions: rationality (*vs* emotionality), social impact, and valence (Tamir *et al.*, 2016). Three similar dimensions—power, sociality and valence—likewise organize neural representation of other people’s traits (Thornton and Mitchell, 2018). These representational spaces not only help people make sense of these social domains, they also allow for automatic prediction of the social future. Both behavioral and neuroimaging research has demonstrated that the dimensions people use to make sense of each other’s thoughts and feelings also lend themselves to the sort of proximity-based prediction we observe here for actions (Tamir *et al.*, 2016; Thornton *et al.*, 2019). In the future, linking these different layers of social cognition together may provide additional insight, such as by shedding light on how knowledge of people’s mental states may help to refine longer-term action predictions.

The present results help to bridge the gap between low-level simulation-based accounts of action understanding rooted in mirror neurons, and higher-level abstract simulations and theory-based accounts of social cognition. The model of predictive action coding presented here is predicated on the ability to perceive and identify others’ current actions (although the present results do not necessarily imply conscious recognition). Those current actions serve as the basis for predicting future actions. Insofar as mirror neurons play a role in the direct perception of actions from visual information, the present findings suggest that mirror neurons need not bear the full weight of people’s action understanding. Instead, that basic understanding of actions may be combined with conceptual knowledge about actions to produce deeper foresight into others’ future behavior.

The present data provide generalizable evidence for a predictive coding account of action representation. First, the overall decoding model was significantly accurate in 94% of participants, indicating a high prevalence of the effect. Second, the decoding models were trained and tested across different participants. This not only provides direct evidence of generalization, but also demonstrates that the neural code supporting action representations is conserved across brains. That said, the accuracy of the decoding model was low in an absolute sense. Moreover, our sample was relatively small and non-representative. Future work would benefit from expanding the size and diversity of the participant sample.

Future research should also test the extent to which these findings generalize beyond a single episode of a particular television show. Although videos are a relatively naturalistic stimulus in the context of current fMRI research, there are important differences between watching a carefully curated narrative from a television show and the experiences people have in their everyday lives. Narratives likely follow social scripts and event schemas that might allow people to predict future actions more successfully than in everyday life (Baldassano *et al.*, 2018). Additionally, it is unclear how well insights from Sherlock, in particular, can be generalized to other narratives. For example, not all possible actions were presented with the video, or were all possible variations of each action. Examination of other naturalistic fMRI data sets with the same model tested here may help to address these generalizability concerns.

The ACT-FAST dimensions are not the only things that people know about actions. It goes without saying that people could describe more than six properties of actions. In the same way, the ACT-FAST is almost certainly not the whole story when it comes to action prediction. In recent work we found that these dimensions partially—but not completely—mediate the accuracy of people’s explicit action predictions (Thornton and Tamir, 2019a). However, one of the reasons that ACT-FAST is a useful representational space is because it oversimplifies the complexity of actions. With the 332 possible actions we consider here, representing every single transition separately would entail remembering over 100 000 different values. Representing the likelihood every three-action sequence would require 12 billion values. This exponential explosion is computationally intractable. Representing actions in a low-dimensional space like ACT-FAST provides a more efficient alternative, while still retaining accuracy. Moreover, representing actions on ACT-FAST dimensions actually enhanced predictability, relative to considering each action in isolation (see Supplementary data).

This study joins a growing body of literature to suggest that brains are organized around the goal of prediction (Hohwy *et al.*, 2008; Friston and Kiebel, 2009; Vuust *et al.*, 2009; Barrett and Simmons, 2015). Although our findings cannot examine the activity of individual neurons, or does it focus on the prediction errors, it suggests that the very way people encode actions supports automatic prediction of future actions. This goal may be particularly impactful for a brain as it engages with the social world (Kilner *et al.*, 2007; Koster-Hale and Saxe, 2013; Tamir and Thornton, 2018; Thornton *et al.*, 2019). Here we provide evidence that the brain automatically predicts others’ actions by encoding them on meaningful psychological dimensions. The way we perceive actions in the present allows us to anticipate others’ future actions before they happen.

## Supplementary Material

nsaa126_Supp

## References

[R1] Abbott, V., Black, J.B., Smith, E.E. (1985). The representation of scripts in memory. *Journal of Memory and Language*, 24, 179–99.

[R2] Baldassano, C., Hasson, U., Norman, K.A. (2018). Representation of real-world event schemas during narrative perception. *Journal of Neuroscience*, 38, 9689–99. 30249790 10.1523/JNEUROSCI.0251-18.2018 PMC6222059

[R3] Barrett, L.F., Bar, M. (2009). See it with feeling: affective predictions during object perception. *Philosophical Transactions of the Royal Society B: Biological Sciences*, 364, 1325–34. 10.1098/rstb.2008.0312 PMC2666711 19528014

[R4] Barrett, L.F., Simmons, W.K. (2015). Interoceptive predictions in the brain. *Nature Reviews. Neuroscience*, 16, 419. 10.1038/nrn3950 PMC4731102 26016744

[R5] Chen, J., Leong, Y.C., Honey, C.J., et al. (2017). Shared memories reveal shared structure in neural activity across individuals. *Nature Neuroscience*, 20, 115–25. 27918531 10.1038/nn.4450 PMC5191958

[R6] Frijda, N.H. (2004). Emotions and action. In: *Feelings and Emotions: The Amsterdam Symposium*, Cambridge, UK: Cambridge University Press, 158–73.

[R7] Friston, K. (2010). The free-energy principle: a unified brain theory? *Nature Reviews Neuroscience*, 11, 127–38. 20068583 10.1038/nrn2787

[R8] Friston, K. (2012). The history of the future of the Bayesian brain. *NeuroImage*, 62, 1230–33. 22023743 10.1016/j.neuroimage.2011.10.004 PMC3480649

[R9] Friston, K., Kiebel, S. (2009). Predictive coding under the free-energy principle. *Philosophical Transactions of the Royal Society B: Biological Sciences*, 364, 1211–21. 10.1098/rstb.2008.0300 PMC2666703 19528002

[R10] Gibson, J.J. (2014). *The Ecological Approach to Visual Perception: Classic Edition*. New York, NY: Psychology Press.

[R11] Gilbert, D.T., Malone, P.S. (1995). The Correspondence Bias. *Psychological Bulletin*, 117, 21–38. 7870861 10.1037/0033-2909.117.1.21

[R12] Hohwy, J., Roepstorff, A., Friston, K. (2008). Predictive coding explains binocular rivalry: an epistemological review. *Cognition*, 108, 687–701. 18649876 10.1016/j.cognition.2008.05.010

[R13] Keysers, C., Gazzola, V. (2010). Social neuroscience: mirror neurons recorded in humans. *Current Biology*, 20, R353–54. 21749952 10.1016/j.cub.2010.03.013

[R14] Kilner, J.M., Friston, K.J., Frith, C.D. (2007). Predictive coding: an account of the mirror neuron system. *Cognitive Processing*, 8, 159–66. 17429704 10.1007/s10339-007-0170-2 PMC2649419

[R15] Knill, D.C., Pouget, A. (2004). The Bayesian brain: the role of uncertainty in neural coding and computation. *Trends in Neurosciences*, 27, 712–19. 15541511 10.1016/j.tins.2004.10.007

[R16] Koster-Hale, J., Saxe, R. (2013). Theory of mind: a neural prediction problem. *Neuron*, 79, 836–48. 24012000 10.1016/j.neuron.2013.08.020 PMC4041537

[R17] Litman, L., Robinson, J., Abberbock, T. (2017). TurkPrime. com: a versatile crowdsourcing data acquisition platform for the behavioral sciences. *Behavior Research Methods*, 49, 433–42. 27071389 10.3758/s13428-016-0727-z PMC5405057

[R18] Maranesi, M., Livi, A., Fogassi, L., et al. (2014). Mirror neuron activation prior to action observation in a predictable context. *Journal of Neuroscience*, 34, 14827–32. 25378150 10.1523/JNEUROSCI.2705-14.2014 PMC6608372

[R19] McIntosh, A.R., Bookstein, F.L., Haxby, J.V., et al. (1996). Spatial pattern analysis of functional brain images using partial least squares. *Neuroimage*, 3, 143–57. 9345485 10.1006/nimg.1996.0016

[R20] Monfort, M., Zhou, B., Bargal, S.A., et al. (2018). Moments in Time Dataset: one million videos for event understanding. *arXiv Preprint*, *arXiv:1801.03150*. 10.1109/TPAMI.2019.2901464 30802849

[R21] Oosterhof, N.N., Tipper, S.P., Downing, P.E. (2013). Crossmodal and action-specific: neuroimaging the human mirror neuron system. *Trends in Cognitive Sciences*, 17, 311–8. 23746574 10.1016/j.tics.2013.04.012

[R0021a] Rao, R.P., Ballard, D.H. (1999). Predictive coding in the visual cortex: a functional interpretation of some extra-classical receptive-field effects. *Nature neuroscience*, 2(1), 79–87. 10195184 10.1038/4580

[R22] Rizzolatti, G., Craighero, L. (2004). The mirror-neuron system. *Annual Review of Neuroscience*, 27, 169–92. 10.1146/annurev.neuro.27.070203.144230 15217330

[R23] Saffran, J.R., Aslin, R.N., Newport, E.L. (1996). Statistical learning by 8-month-old infants. *Science*, 274, 1926–8. 8943209 10.1126/science.274.5294.1926

[R24] Saygin, A.P., Chaminade, T., Ishiguro, H., et al. (2011). The thing that should not be: predictive coding and the uncanny valley in perceiving human and humanoid robot actions. *Social Cognitive and Affective Neuroscience*, 7, 413–22. 21515639 10.1093/scan/nsr025 PMC3324571

[R25] Tamir, D.I., Thornton, M.A. (2018). Modeling the predictive social mind. *Trends in Cognitive Sciences*, 22, 201–12. 29361382 10.1016/j.tics.2017.12.005 PMC5828990

[R26] Tamir, D.I., Thornton, M.A., Contreras, J.M., Mitchell, J.P. (2016). Neural evidence that three dimensions organize mental state representation: rationality, social impact, and valence. *Proceedings of the National Academy of Sciences*, 113(1), 194–9. 10.1073/pnas.1511905112 PMC4711847 26621704

[R27] Theriault, J., Young, L. (2017). Social prediction in the theory of mind network.

[R28] Thornton, M.A., Mitchell, J.P. (2018). Theories of person perception predict patterns of neural activity during mentalizing. *Cerebral Cortex*, 28, 3505–20. 28968854 10.1093/cercor/bhx216

[R30] Thornton, M.A., Tamir, D.I. (2019a). Perceptions accurately predict the transitional probabilities between actions. *PsyArXiv*.

[R31] Thornton, M.A., Tamir, D.I. (2019b). Six dimensions describe action understanding: the ACT-FASTaxonomy. *PsyArXiv*. 10.1037/pspa0000286 PMC8901456 34591540

[R32] Thornton, M.A., Weaverdyck, M.E., Tamir, D.I. (2019). The social brain automatically predicts others’ future mental states. *Journal of Neuroscience*, 39, 140–8. 30389840 10.1523/JNEUROSCI.1431-18.2018 PMC6325264

[R33] Vuust, P., Ostergaard, L., Pallesen, K.J., et al. (2009). Predictive coding of music–brain responses to rhythmic incongruity. *Cortex*, 45, 80–92. 19054506 10.1016/j.cortex.2008.05.014

[R34] Zhou, B., Andonian, A., Torralba, A. (2017). Temporal relational reasoning in videos. *arXiv Preprint*, *arXiv:1711.08496*.

