# Supplementary material for: Perceiving actions before they happen: psychological dimensions scaffold neural action prediction
Source: Soc Cogn Affect Neurosci. 2020 Sep 28;16(8):807–15. doi: 10.1093/scan/nsaa126 (PMC8343568; doi:10.1093/scan/nsaa126)
Supplement: nsaa126_Supp [file nsaa126_supp.zip › Supplementary_material.docx]

**Supplementary Material**

**Autocorrelation of ACT-FAST dimensions**
 In addition to our neural analyses, we conducted one analysis using just the action annotations of *Sherlock* and the human ratings. In this analysis, we computed time series for each of the six ACT-FAST dimensions by taking probability-weighted averaged of the action’s coordinates over time, as described below. However, we also repeated this process using actions which had been randomly permuted on the ACT-FAST dimensions. The resulting dimensions spanned the action space in a statistically similar way, but without any inherent meaning. We then computed the (lag 1) autocorrelation of these times series, comparing the actual ACT-FAST time series to their permuted versions. Due to the way the annotations were computed (every 2 TRs sharing the same action label) we computed autocorrelation using only the odd TRs. To the extent that the ACT-FAST autocorrelations were higher than those of the permuted dimensions, this would indicate that the taxonomy is particularly well-suited to capturing natural action dynamics.

Across the ACT-FAST dimensions, the mean autocorrelation was *r* = 55. This was higher than the autocorrelation in 95.72% of the 5000 equivalent sets of dimensions generated through permutation (mean *r* = .51). This suggests that – apart from whether or not the brain encodes them during action perception – the ACT-FAST dimensions are a particularly good basis set for describing the natural dynamics of action over time.

We also examined a related question: whether representing actions as coordinates in ACT-FAST space enhances predictability, relative to representing each action discretely? To answer this question, we applied the approach above to compute the autocorrelations in the probabilities of all 332 actions over the course of *Sherlock*. The average autocorrelation of an individual action was *r* = .36. This indicates that actions do indeed predict themselves over time. However, the average autocorrelation of the ACT-FAST dimensions (r = .55) was substantially higher (54%) than that of the individual actions. Indeed, every dimension of the ACT-FAST was more predictable than the vast majority of individual actions: Abstraction (93%), Creation (92%), Tradition (86%), Food (86%), Animacy (89%), and Spiritualism (89%). These findings illustrate how the representing actions using ACT-FAST is not only parsimonious, but also increases predictability.

**Supplementary Figures**


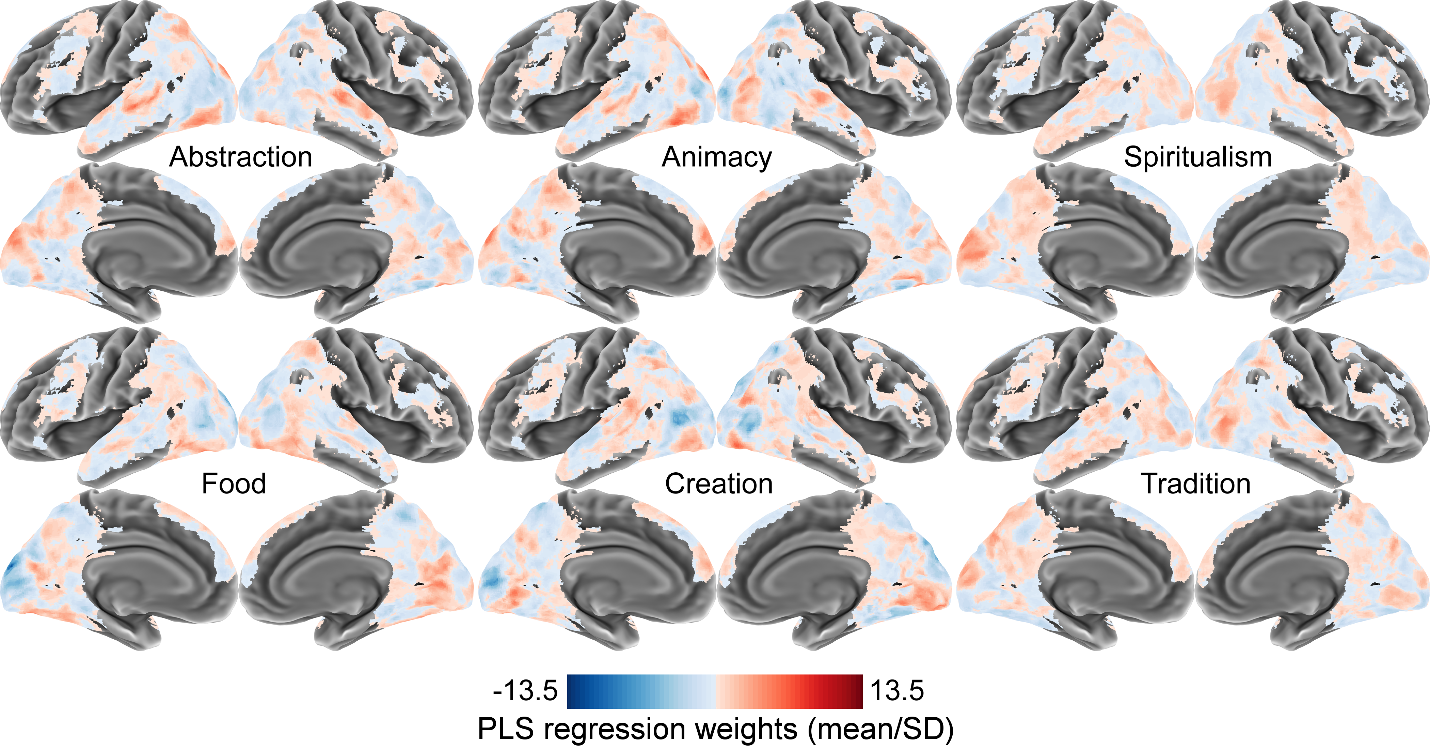


**Figure S1. Regions contributing to ACT-FAST decoding.** PLS regressions were trained to decode ACT-FAST coordinates from patterns of brain activity within action sensitive regions. Here we visualize the beta weights learned by these regressions. The positive versus negative signs of the weights indicate which pole of each dimension each voxel was associated with. For example, red = abstract and blue = concrete for the Abstraction dimension. To provide a sense of the consistency of these weights across different participants and parts of Sherlock, we display them here in terms of a pseudo Cohen’s *d*: that is, the average coefficient across cross-validation folds, divided by its standard deviation (this is not a true Cohen’s d, since the cross-validation folds are not independent, and so underestimate the standard deviation). The results indicate that consistent spatial patterns encode the ACT-FAST dimensions across different participants brains.
