# Supplementary figures and images for: Perceiving actions before they happen: psychological dimensions scaffold neural action prediction
Source: Soc Cogn Affect Neurosci. 2020 Sep 28;16(8):807–15. doi: 10.1093/scan/nsaa126 (PMC8343568; doi:10.1093/scan/nsaa126)

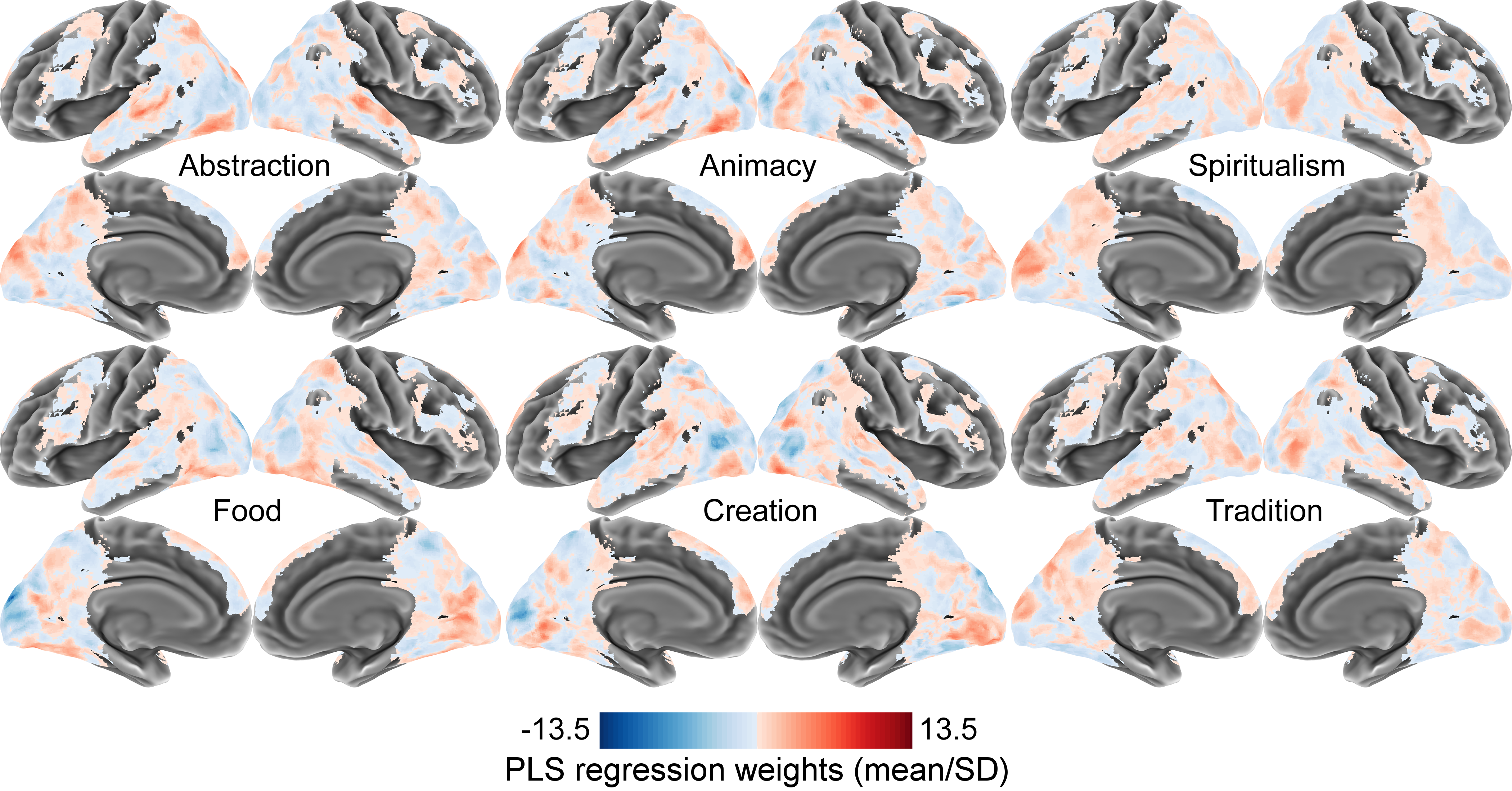

Supplement: nsaa126_Supp [file nsaa126_supp.zip › Figure_S1.tif]
